# Associations of dietary patterns with hypertension among adults in Jilin Province, China: a structural equation modelling approach

**DOI:** 10.1017/S1368980018003129

**Published:** 2018-12-27

**Authors:** Junsen Ye, Yaogai Lv, Zhongmin Li, Yan Yao, Lina Jin

**Affiliations:** 1 Epidemiology and Biostatistics, School of Public Health, Jilin University, No. 1163 Xinmin Street, Changchun, Jilin 130021, People’s Republic of China; 2 The Second Affiliated Hospital (Jiande Branch), Department of Cardiology, Zhejiang University School of Medicine, No. 599 Yanzhou Street, Jiande, Zhejiang 311600, People’s Republic China

**Keywords:** Hypertension, Dietary patterns, Factor analysis, Structural equation modelling

## Abstract

**Objective:**

To explore the direct and indirect associations of dietary patterns with hypertension using structural equation modelling (SEM).

**Design:**

Factor analysis with varimax rotation was used to classify different dietary patterns and SEM was employed to investigate the associations of dietary patterns with hypertension. Total cholesterol to HDL-cholesterol (TC:HDL-C) ratio and LDL-cholesterol to HDL-cholesterol (LDL-C:HDL-C) ratio were used as observed indicator variables of the lipid latent variable. Waist circumference, body fat percentage and BMI, which were associated with hypertension, were used as observed indicator variables of the obesity latent variable.

**Setting:**

International Chronic Disease Cohort (ICDC) that began in 2005 with the purpose of describing the frequency and determinants of chronic diseases in Jilin Province, China.

**Participants:**

A total of 1492 adults (40–79 years) were enrolled in the baseline study from August 2010 to August 2011.

**Results:**

Hypertension prevalence in our study population was 34·9 %. It was found that the wine pattern, condiment pattern, obesity latent variable, lipid latent variable, glucose, age and family history of hypertension were factors that had an association with hypertension via SEM, and the corresponding coefficients were 0·056, 0·011, 0·230, 0·281, 0·098, 0·232 and 0·116, respectively.

**Conclusions:**

The wine pattern and lipid latent variable had positive direct associations with hypertension. The condiment pattern had a positive indirect association with hypertension via the obesity latent variable. The vegetables pattern, modern pattern and snack pattern were not associated with hypertension.

Hypertension is one of the leading global public health problems, affecting more than 1 billion people worldwide, and is also believed a major risk factor for developing CVD, stroke, etc. Thus, it has been identified as an important risk factor for mortality and is the first-ranked risk factor for total burden of disease worldwide^(^ ^1^ ^,^ ^2^ ^)^. Many studies have been put forward to investigate hypertension in the literature^(^ ^3^ ^,^ ^4^ ^)^.

It is well established that hypertension is associated with many factors, such as alcohol consumption, exercise, genetic factors and especially dietary factors^(^ ^5^ ^,^ ^6^ ^)^. Over the past few decades, a number of studies have emerged concerning the associations between dietary factors and hypertension^(^ ^7^ ^)^. However, there are few works performing the construction of the factors/patterns and studying the associations at same time using structural equation modelling (SEM). Most of them have focused on the associations between a single food or nutrient and hypertension^(^ ^1^ ^)^. In addition, with the rapid development of social economy, the variety of foods has become more diversified and so the interactions between various foods and nutrients have become more complex accordingly. Therefore, these analyses have revealed limited impacts of diet on hypertension^(^ ^8^ ^)^. Further research is needed to establish the effect of dietary patterns on blood pressure in different cultures other than those identified in the above brief review.

Fortunately, the analysis of dietary patterns, which represent the whole diet and a complex integration of foods and nutrients, was proposed to overcome the limitations mentioned above^(^ ^9^ ^)^. Further, the investigation of the overall effects of diet on human health is also allowed with dietary patterns, by taking account of the complex interactions among foods and nutrients consumed^(^ ^10^ ^)^. And the dietary patterns consumed have been indicated to play an important role in the development of hypertension as well^(^ ^11^ ^)^.

Generally, logistic regression has been used to explore the association of dietary patterns with hypertension. On the other hand, SEM allows for multiple linear equations, including direct and indirect associations, and for latent variables, features not allowed by traditional regression methods^(^ ^10^ ^)^. In the present study, we aimed to use SEM to explore the direct and indirect associations of dietary patterns with hypertension.

## Methods

### Study population

Data were obtained from the International Chronic Disease Cohort (ICDC) that began in 2005 with the purpose of describing the frequency and determinants of chronic diseases in Jilin Province, China. A total of 1492 participants (aged 40–79 years) was enrolled in the baseline study from August 2010 to August 2011. For analysis purposes, some individuals were excluded due to missing values for blood pressure. Finally, a total of 1326 participants was included in the present analyses. All procedures in the study involving human subjects were approved by the Research Ethics Committees at the School of Public Health, University of Jilin. All participants provided their written informed consent before data collection in each stage of the study.

### Dietary data collection

Trained investigators from Jilin University visited participants in their homes to collect dietary information using a validated FFQ over the past year. This FFQ was based on the FFQ used in the 2002 China National Nutrition and Health Survey. Because of the low intake of some food items, eighty-nine food items in the FFQ were divided into nineteen food groups based on the role of foods in the diet and nutritional characteristics (for details see the online supplementary material, Supplemental Table 1). Participants were asked to recall the frequency of consumption of individual food items over the previous 12 months. The frequency of food intake was measured using nine categories: never, <12 times/year, 1–3 times/month, 1–2 times/week, 3–4 times/week, 5–6 times/week, 1 time/d, 2 times/d and 3 times/d. The participants also estimated the portion size, using local weight units (1 liang = 50 g) or household measures (cups). Intakes of foods were converted into g/week for data analysis. Our prior investigation of this FFQ found favourable dietary intake estimation characteristics compared with dietary intake estimated by multiple 24 h dietary recalls. The correlation coefficients between the FFQ and means from the 24 h dietary recalls ranged between 0·51 to 0·66 for food groups.

### Identification of dietary patterns

First, the Kaiser–Meyer–Olkin measure of sample adequacy and the Bartlett test of sphericity were used to assess data adequacy for factor analysis. The result of the Kaiser–Meyer–Olkin test was 0·685 and the Bartlett test was significant (*P* <0·001), indicating that factor analysis of the data would be useful. Next, factor analysis with the principal component factor extraction method was conducted using the statistical software package IBM SPSS Statistics version 20.0, and varimax rotation was used to make uncorrelated factors easier and simpler to interpret. The eigenvalue and scree plot were applied to decide which factors remained^(^ ^9^ ^)^. After evaluating the eigenvalues, the scree plot test (for details see the online supplementary material, Supplemental Fig. 1) and interpretability, factors with eigenvalues ≥1·0 were retained.

### Assessment of anthropometric and clinical measures

The study data included demographics (gender, age, etc.), health-related behaviours (smoking, drinking, etc.), anthropometric measurements (height, weight, hypertension, etc.) and laboratory measurements (such as serum cholesterol and TAG). All investigators were trained and followed the same questionnaire instructions.

Well-trained examiners measured the anthropometric indices of participants with light clothing and without shoes. Height was measured to the nearest 0·1 cm and weight was measured to the nearest 0·1 kg using electronic scales. Body fat percentage (FAT) was measured using a body composition measuring device (TANITA BC-600-WH). BMI was calculated by dividing weight (in kilograms) by the square of height (in metres). Waist circumference (WC) was measured to the nearest 0·1 cm at the middle point between the lowest point of the rib cage and the iliac crest.

A mercury sphygmomanometer was used to measure each participant’s blood pressure in the sitting position after a 10 min rest period. The appearance of the first sound was used to define systolic blood pressure (SBP) and the disappearance of the sound was used to define diastolic blood pressure (DBP). Two readings each of SBP and DBP were recorded, and the average of each measurement was used for data analysis. If the first two measurements differed by more than 5 mmHg, additional readings were taken.

Blood samples were obtained from the antecubital vein into anticoagulant tubes containing EDTA in the morning after an overnight fasting period. All collected samples were transported on dry ice at prearranged intervals to the central laboratory. Serum lipids, including total cholesterol (TC), TAG, HDL-cholesterol (HDL-C) and LDL-cholesterol (LDLC), were measured using an auto-analyser (Beckman Synchron LX20). Fasting plasma glucose (FPG) levels were measured using the Bayer Bai Ankang fingertip blood glucose monitoring machine by taking a small drop of blood from a finger on to a strip of paper in the morning after participants had fasted for 10 h or more overnight.

### Hypertension assessment criteria

According to the Seventh Joint National Commission Guidelines, hypertension was defined as resting SBP/DBP ≥140/90 mmHg or current use of antihypertensive medication^(^ ^12^ ^)^.

### Statistical analysis

SEM was used to explore the associations between dietary patterns and hypertension, and was conducted with AMOS version 20.0 for SPSS using the MLR estimation method and the oblique geomin rotation. The MLR estimation method was chosen because it is an iterative estimation procedure that leads to more robust standard error estimates for continuous data following a non-normal multivariate distribution^(^ ^13^ ^)^. The mixed variables of age and history of hypertension were controlled in SEM. We evaluated the model fit using the normed *χ* ^2^ statistic (*χ* ^2^/df), the goodness-of-fit index (GFI), the adjusted goodness-of-fit fit index (AGFI), the normed fit index (NFI), the root-mean-square error of approximation (RMSEA), the comparative fit index (CFI), the Tucker–Lewis index (TLI) and the standardized root-mean-square residual (SRMR). Standardized estimates of regression coefficients, correlation coefficients and indirect effects were reported. Standardized coefficients reflect the degree of change in the outcome variable associated with a standard deviation change in the predictor. Standardized regression coefficients permit comparisons of predictor–outcome relationships across studies in which the variables have been measured using different units of measure. *P* <0·05 was considered to indicate significance in two-sided tests. Acceptable model fit was defined according to the following criteria: *χ* ^2^/df <3, GFI >0·90, AGFI >0·90, NFI >0·90, TLI >0·90, CFI >0·90, SRMR <0·08, RMSEA ≤ 0·05 and 90 % CI <0·08^(^ ^14^ ^,^ ^15^ ^)^.

## Results

The 1326 participants included in our study included 463 hypertensive individuals and 863 non-hypertensive individuals (the overall prevalence of hypertension was 34·9 %), and their demographic and lifestyle characteristics are shown in Table 1. There were significant differences between hypertensive and non-hypertensive participants by age, BMI, WC, FAT, GLU, TC:HDL-C, LDL-C:HDL-C and family history of hypertension, whereas there were no significant differences by gender, educational level, occupation and smoking status.

**Table 1:** Descriptive characteristics, by hypertension status, of participants aged 40–79 years (*n* 1326) from Jilin Province, China, enrolled in the International Chronic Disease Cohort, 2010–2011

Variable	Non-hypertensive (*n* 863)		Hypertensive (*n* 463)		*t*	*P*
	Mean	sd	Mean	sd		
Age (years)	52·9	9·7	57·5	8·8	74·722	<0·001
BMI (kg/m^2^)	23·1	3·2	24·8	3·5	80·023	<0·001
WC (cm)	81·3	8·7	86·1	8·9	89·687	<0·001
FAT (%)	28·9	8·2	31·4	8·6	25·654	<0·001
GLU (mmol/l)	4·9	1·2	5·3	1·4	15·786	<0·001
TC:HDL-C	3·6	1·1	3·9	1·1	17·967	<0·001
LDL-C:HDL-C	2·3	0·9	2·5	0·9	14·086	<0·001
						
	*n*	%	*n*	%	*χ* ^2^	*P*
Gender						
Male	324	37·5	178	38·4	0·104	0·396
Female	539	62·5	285	61·6		
Education level						
Primary school	225	26·1	141	30·5	4·675	0·322
Junior high school	492	57·0	258	55·7		
Senior high school	106	12·3	50	10·8		
Other^*^	40	4·6	14	3·0		
Occupation						
Mental labour	47	5·4	32	6·9	4·282	0·118
Manual labour	774	89·7	398	86·0		
Other†	42	4·9	33	7·1		
Smoking						
Yes	76	8·8	33	7·1	1·878	0·391
No	517	59·9	293	63·3		
Abstinence	270	31·3	137	29·6		
Family history of hypertension						
Yes	86	9·9	81	17·5	15·519	<0·001
No	777	90·1	382	82·5		

WC, waist circumference; FAT, body fat percentage; GLU, glucose; TC, total cholesterol; HDL-C, HDL-cholesterol; LDL-C, LDL-cholesterol.

* Other’ includes illiteracy and college degree or above.

† Other’ includes unemployed and retired people.

The factor loading matrix is shown in Table 2. Five major dietary patterns were identified (explaining 50·58 % of the total variance), namely ‘wine pattern’, ‘vegetables pattern’, ‘condiment pattern’, ‘modern pattern’ and ‘snack pattern’, which explained 16·73, 11·18, 9·35, 7·38 and 5·94 % of the dietary intake variance, respectively.

**Table 2:** Factor loading matrix for major dietary patterns identified among participants aged 40–79 years (*n* 1326) from Jilin Province, China, enrolled in the International Chronic Disease Cohort, 2010–2011

Food	Wine pattern	Vegetables pattern	Condiment pattern	Modern pattern	Snack pattern
White wine	**0·957**	0·045	−0·031	0·003	0·099
Beer	**0·957**	0·056	−0·049	0·010	0·101
Seasoning	**0·522**	0·236	0·120	0·069	−0·134
Cooked vegetables	0·154	**0·746**	−0·024	0·188	0·093
Raw vegetables	0·130	**0·735**	0·007	0·040	0·110
Pickles	0·099	**0·706**	−0·111	−0·061	0·065
Rice	−0·115	**0·365**	0·213	0·056	−0·141
Salt	−0·152	−0·034	**0·866**	0·068	0·015
Oil	0·035	0·086	**0·836**	0·069	−0·064
Sugar	0·179	−0·061	**0·607**	0·056	0·168
Lean meats	0·120	−0·067	0·025	**0·768**	−0·021
Drinks	−0·022	−0·005	0·059	**0·566**	0·113
Pasta	0·010	0·181	0·009	**0·489**	0·221
Eggs	0·003	0·360	−0·011	**0·403**	0·103
Fish	−0·022	0·033	0·073	**0·387**	0·014
Soya products	0·194	0·348	−0·294	**0·362**	−0·048
Milk	0·095	−0·012	−0·045	0·141	**0·683**
Biscuits	−0·089	−0·002	0·062	0·053	**0·682**
Fruits	0·081	0·329	0·109	0·130	**0·538**

In considering the number of factors to retain, we evaluated eigenvalues (>1), the scree plot and the interpretability of the factors to determine which set of factors could most meaningfully describe distinct food patterns. Items were retained in a factor if they had an absolute correlation of ≥0·30 with that factor (indicated in bold font).

**Fig. 1:**
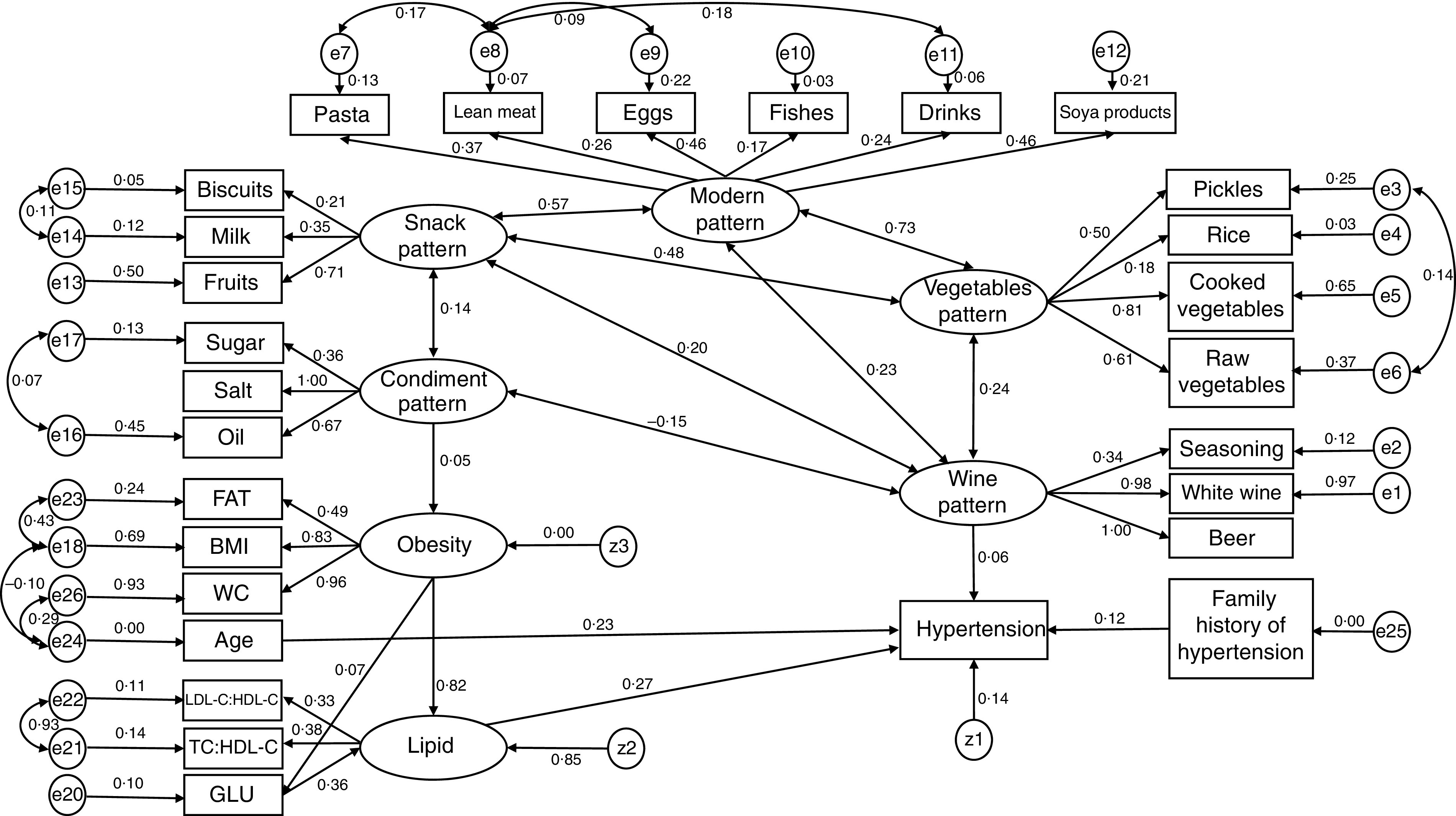
Structural equation model with standardized estimates for the relationship between dietary patterns and hypertension among participants aged 40–79 years (*n* 1326) from Jilin Province, China, enrolled in the International Chronic Disease Cohort, 2010–2011 (FAT, body fat percentage; WC, waist circumference; LDL-C, LDL-cholesterol; HDL-C, HDL-cholesterol; GLU, glucose; *e_i_* (*i* = 1, …, 26) and *z_j_* (*j* = 1, …, 3) are residuals)

After evaluating the eigenvalues, the scree plot test and interpretability, factors with eigenvalues ≥1·0 were retained, and individual food items with absolute factor loadings ≥0·3 were considered to contribute significantly to the pattern. We choose the larger loading when different dietary patterns included the same foods that had loadings ≥0·3. Thus, the detailed foods of the five dietary patterns mentioned above are: wine pattern (greater intake of schnapps, beer and seasoning), vegetables pattern (greater intake of cooked vegetables, raw vegetables, pickles and rice), condiment pattern (greater intake of salt, oil and sugar), modern pattern (greater intake of lean meats, drinks, pasta, eggs, fish and soya products) and snack pattern (greater intake of milk, biscuits and fruits). BMI, WC, FAT, GLU, TC:HDL-C and LDL-C:HDL-C were added into the SEM, because those variables might be intermediate steps in the causal biological pathway for the effect of dietary patterns on hypertension. Considering that BMI, WC and FAT represent obesity indicators, a latent variable named ‘obesity latent variable’ was constructed. Moreover, a potential variable called ‘lipid latent variable’ was constructed to reflect TC:HDL-C and LDL-C:HDL-C. Figure 1 shows the SEM diagram with standardized estimates for the relationships between dietary patterns and hypertension and risk factors. The one-sided arrows from the lipid latent variable and wine pattern to hypertension represent the regression coefficients; whereas the arrows from the obesity and lipid latent variables to FAT, BMI and WC, and to LDL-C:HDL-C and TC:HDL-C, respectively, indicate the standardized factor loadings of the measured variables. The two-sided arrows represent the correlation coefficients between dietary patterns. The residual correlation coefficients between outcome variables were omitted for simplicity. The first model was tested to explore the direct and indirect associations between dietary patterns and hypertension; its goodness-of-fit indices indicated an unacceptable fit (*χ* ^2^/df = 5·966, GFI = 0·899, AGFI = 0·880, NFI = 0·842, TLI = 0·833, CFI = 0·865, SRMR = 0·055, RMSEA = 0·065 and 90 % CI 0·061, 0·069). In addition to being explained by latent variables, some of observed variables may be explained by residuals and they have some degree of correlation. The final model was obtained by decreasing the *χ* ^2^ values (or optimizing the modification index) and increasing residual correlations. In contrast to the first model, the goodness-of-fit indices of the final model indicated an acceptable fit (*χ* ^2^/df = 2·502, GFI = 0·957, AGFI = 0·947, NFI = 0·939, TLI = 0·957, CFI = 0·962, SRMR= 0·045, RMSEA = 0·034 and 90 % CI 0·031, 0·037; Table 3).

**Table 3:** Parameter estimates and model fit from the structural equation modelling of dietary patterns and hypertension among participants aged 40–79 years (*n* 1326) from Jilin Province, China, enrolled in the International Chronic Disease Cohort, 2010–2011

Item			Standardized estimate	*P*
Direct effects of variables				
Wine pattern	→	Hypertension	0·057	0·028
Lipid latent variable	→	Hypertension	0·281	<0·001
Age	→	Hypertension	0·231	<0·001
Family history of hypertension	→	Hypertension	0·162	<0·001
Condiment pattern	→	Obesity latent variable	0·054	0·033
Obesity latent variable	→	GLU	0·073	0·006
Obesity latent variable	→	Lipid latent variable	0·822	<0·001
GLU	→	Lipid latent variable	0·359	<0·001
Factor loadings of obesity latent variable				
Obesity	→	BMI	0·826	<0·001
Obesity	→	WC	0·963	<0·001
Obesity	→	FAT	0·488	<0·001
Factor loadings of lipid latent variable				
Lipid	→	TC:HDL-C	0·382	<0·001
Lipid	→	LDL-C:HDL-C	0·330	<0·001
Correlation coefficients				
Vegetables pattern	↔	Snack pattern	0·483	<0·001
Wine pattern	↔	Vegetables pattern	0·239	<0·001
Condiment pattern	↔	Snack pattern	0·141	<0·001
Vegetables pattern	↔	Modern pattern	0·732	<0·001
Snack pattern	↔	Modern pattern	0·574	<0·001
Wine patterns	↔	Modern pattern	0·234	<0·001
Wine pattern	↔	Snack pattern	0·198	<0·001
Wine pattern	↔	Condiment pattern	−0·153	<0·001
Model fit				
*χ* ^2^/df			2·502	<0·001
NFI			0·939	
TLI			0·957	
CFI			0·962	
GFI			0·957	
AGFI			0·947	
SRMR			0·045	
RMSEA			0·034	

GLU, glucose; WC, waist circumference; FAT, body fat percentage; TC, total cholesterol; HDL-C, HDL-cholesterol; LDL-C, LDL-cholesterol; *χ* ^2^/df, normed *χ* ^2^; NFI normed fit index; TLI, Tucker–Lewis index; CFI, comparative fit index; GFI, goodness-of-fit index; AGFI, adjusted goodness-of-fit index; SRMR, standardized root-mean-square residual; RMSEA, root-mean-square error of approximation.

In general, all indices suggested that the presented final model fitted the data reasonably (Tables 3 and 4). The vegetables pattern, modern pattern and snack pattern were not associated with hypertension (*P* >0·05). The wine pattern, obesity latent variable, lipid latent variable, GLU, age and family history of hypertension had positive direct associations with hypertension (*P* <0·05), and the condiment pattern had a positive direct effect on the obesity latent variable (*β* = 0·053, *P* = 0·033). Moreover, the obesity latent variable was an important mediator in the relationship between the condiment pattern and hypertension.

**Table 4:** Total, direct and indirect effects of independent variables on hypertension among participants aged 40–79 years (*n* 1326) from Jilin Province, China, enrolled in the International Chronic Disease Cohort, 2010–2011

Item	Direct effects	Indirect effects	Total effects
Condiment pattern	–	0·011	0·011
Wine pattern	0·056	–	0·056
Obesity latent variable	–	0·230	0·230
GLU	–	0·098	0·098
Lipid latent variable	0·281	–	0·281
Age	0·232	–	0·232
Family history of hypertension	0·116	–	0·116

GLU, glucose.

## Discussion

It is believed that dietary patterns have important impacts on the development of hypertension^(^ ^16^ ^–^ ^19^ ^)^; however, there are few works that have performed the construction of the factors/patterns and studied the associations at same time by using SEM^(^ ^20^ ^–^ ^22^ ^)^. In the present study, we explored the direct and indirect associations of dietary patterns with hypertension using SEM. The wine pattern, which was characterized by high loadings from schnapps, beer and sauces (seasoning), showed a direct positive association with hypertension. The condiment pattern, which was characterized by high loadings from salt, oil and sugar, showed an indirect positive association with hypertension via the obesity latent variable.

The wine pattern was found to be a risk factor for hypertension, which was consistent with previous studies^(^ ^11^ ^,^ ^20^ ^)^. A lot of published studies have demonstrated that drinking alcohol is a risk factor for hypertension^(^ ^5^ ^,^ ^23^ ^)^. Several possible mechanisms have been proposed. On one hand, alcohol may affect blood pressure by stimulating the renal angiotensin–aldosterone system and increasing vascular reactivity, increasing cortisol levels, increasing vascular reactivity due to increase in intracellular Ca levels and stimulating the endothelium to release vasoconstrictors^(^ ^24^ ^)^; on another hand, alcohol may lead to an increase in blood pressure by influencing heart rate and insulin sensitivity^(^ ^24^ ^)^.

Further, the condiment pattern was found to be an indirect risk factor for hypertension. First, salt intake has been acknowledged as a risk factor for hypertension, where high Na intake contributes to increase TAG, HDL hypercholesterolaemia, WC, BMI, abdominal obesity, blood pressure and body fat percentage^(^ ^25^ ^,^ ^26^ ^)^. Second, salty foods also stimulate the brain’s reward response, and the increased energy intake augments the incidence of overeating and obesity-related diseases^(^ ^27^ ^,^ ^28^ ^)^. Third, foods high in fat and sugar, which define the high-fat/high-sucrose diet^(^ ^29^ ^)^, are well established as comfort foods. In addition, habitual use of these foods, perhaps stimulated by abnormally elevated concentrations of cortisol as a consequence of underlying stressors, results in abdominal obesity and metabolic syndrome^(^ ^30^ ^)^.

It is noteworthy that the vegetables pattern, modern pattern and snack pattern were not associated with hypertension in our analysis, which is inconsistent with previous studies^(^ ^7^ ^)^. Some studies have also indicated that a dietary pattern rich in fruits and vegetables could decrease the risk of hypertension^(^ ^31^ ^,^ ^32^ ^)^. However, Chinese tend to consume more cooked vegetables, which may result in the loss of some antioxidant content^(^ ^33^ ^)^. Furthermore, the middle-aged population in Jilin Province often prefers to eat kimchi and vegetables with sauce.

In general, the findings in our study imply that not only reducing one’s alcohol consumption might be beneficial to prevent and control hypertension. Reducing one’s intakes of salt and foods high in fat and sugar might also bring benefits to prevent and control hypertension among adults in Jilin Province, considering that the condiment pattern was also associated with obesity disturbances and then had an indirect association with hypertension.

Some limitations of our study should be noted. First, the results were from a cross-sectional study in Jilin Province, which might limit the ability to generalize the results to other areas. Second, other confounders that might have impacts on hypertension, such as physical activity, sleeping time, etc., were not considered in the present study, which might have some slight effects on our results. Third is the memory bias that may occur when a self-reported and long-term dietary assessment method such as the semi-quantitative FFQ is applied for older adults, as well as other limitations associated with the FFQ. Finally, the subjectivity in factor analysis modelling, considering that a predetermined factor loading cut-off (0·30) was applied, might have slight impacts on the results. However, data from other populations can also be analysed using dietary patterns and SEM.

## Conclusions

The wine pattern and lipid latent variable had positive direct associations with hypertension. The condiment pattern had a positive indirect association with hypertension via the obesity latent variable. The vegetables pattern, modern pattern and snack pattern were not associated with hypertension.

## Supplementary material

For supplementary material accompanying this paper visit https://doi.org/10.1017/S1368980018003129. click here to view supplementary material
