# Supplementary material for: Associations of dietary patterns with hypertension among adults in Jilin Province, China: a structural equation modelling approach
Source: Public Health Nutr. 2018 Dec 27;22(6):1048–55. doi: 10.1017/S1368980018003129 (PMC6536898; doi:10.1017/S1368980018003129)
Supplement: Supplementary file 1 [file S1368980018003129sup001.docx]

**Online Supplementary Material to**

**Associations between Dietary Patterns and Hypertension among Adults in Jilin Province: A Structural Equation Modelling Approach**

1. **Food group and Food items**

According to the similarities in food nutrient profiles and culinary usage, the 89 food items in the FFQ were categorized into 19 food groups to reduce the complexity of the data (Table 1). This regrouping of food items was based on the previous study with modification.

Table 1 Food group and food items used in dietary pattern analysis

| Food group | Food items |
| --- | --- |
| White wine | White wine |
| Beer | Beer |
| Seasoning | Sichuan Pepper, soy sauce, vinegar, aginomoto, cooking wine |
| Cooked vegetables | Cucumber, eggplant, leek, garlic moss, celery, spinach, beans, cabbage, turnip, turnip, carrot, pumpkin, seaweed, seaweed, pepper, potato |
| Raw vegetables | Lettuce, cucumber, tomato, cilantro |
| Pickles | Spicy cabbage, radish salted vegetables, turnip vegetables, mustard, pickles, pickles, pickled radishes |
| Rice | Rice, porridge |
| Salt | Salt |
| Oil | Vegetable oil, animal oil |
| Sugar | Sugar |
| Lean meats | Beef, pork, mutton, dog meat, chicken, duck |
| Drinks | Carbonated drinks, juice |
| Pasta | Corn flour, soba noodles, steamed bread, noodles, oil cake, dumplings, bread, and cake |
| Eggs | Eggs, duck eggs, goose eggs, quail eggs |
| Fishes | Chinese mackerel, ba fish, herring, sword fish, yellow croaker, mingtai fish, carp, carp, loach, squid, shrimp, crab |
| Soy products | Tofu, soy milk, bean sprouts |
| Milk | Milk, ice cream |
| Biscuits | Cookies, chocolate |
| Fruits | Melon, grape, watermelon, apple, pear, persimmon, peach, banana, orange |

1. **scree plot**

After evaluating the eigenvalues, the scree plot test (Figure 1) and interpretability, factors with eigenvalues ≥1.0 were retained, and individual food items with a factor loading ≥0.3 were considered to significantly contribute to the pattern in this study.

Figure


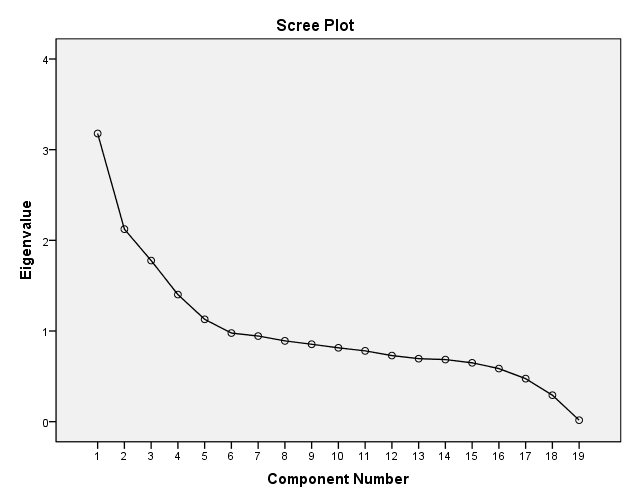


Figure 1 The screen plot test
